# Design of bilayer tablets using modified *Dioscorea* starches as novel excipients for immediate and sustained release of aceclofenac sodium

**DOI:** 10.3389/fphar.2014.00294

**Published:** 2015-01-12

**Authors:** Adenike Okunlola

**Affiliations:** Department of Pharmaceutics and Industrial Pharmacy, Faculty of Pharmacy, University of IbadanIbadan, Nigeria

**Keywords:** aceclofenac sodium, acid-modification, bilayer tablets, carboxymethylation, *Dioscorea* starches, excipients

## Abstract

Bilayer tablets of aceclofenac sodium were developed using carboxymethylated white yam (*Dioscorea rotundata*) starch (CWY) for a fast release layer (2.5, 5.0, and 7.5% w/w), and acid-hydrolyzed bitter yam (*Dioscorea dumetorum*) starch (ABY) for a sustaining layer (27% w/w). Sodium starch glycolate (SSG) and hydroxypropyl methyl cellulose (HPMC) were used as standards. The starches were characterized using Fourier Transform Infrared spectroscopy (FT-IR), particle size, swelling power, densities and flow analyses. Mechanical properties of the tablets were evaluated using crushing strength and friability while release properties were evaluated using disintegration and dissolution times. Distinctive fingerprint differences between the native and modified starches were revealed by FT-IR. Carboxymethylation produced starches of significantly *(p* < 0.05) higher swelling and flow properties while acid-modification produced starches of higher compressibility. Bilayer tablets containing ABY had significantly higher crushing strength and lower friability values (*p* < 0.05) than those containing HPMC. Crushing strength increased while friability values decreased with increase in CWY. Generally tablets containing the modified *Dioscorea* starches gave faster (*p* < 0.05) disintegration times and produced an initial burst release to provide the loading dose of the drug from the immediate-release layer followed by sustained release (300 ± 7.56–450 ± 11.55 min). The correlation coefficient (*R*^2^) and chi-square (χ^2^) test were employed as error analysis methods to determine the best-fitting drug release kinetic equations. *In vitro* dissolution kinetics generally followed the Higuchi and Hixson-Crowell models via a non-Fickian diffusion-controlled release. Carboxymethylated white yam starch and acid-modified bitter yam starch could serve as cheaper alternative excipients in bilayer tablet formulations for immediate and sustained release of drugs respectively, particularly where high mechanical strength is required.

## Introduction

The cost of developing a new drug molecule is usually high; efforts are therefore directed at the development of new dosage forms for existing drugs with improved safety and efficacy as well as reduced dosing frequency and cost. Tablets are the most preferred dosage forms for oral route of drug administration due to their convenience, ease of dosing administration, accuracy of dosage, flexibility in formulation, pain avoidance and patient compliance (Banker and Anderson, 1986; Ansel et al., 2011). Conventional monolayer tablets however have the limitations of frequent dosing and unpredictable absorption window that may result in wide range of fluctuation in drug concentration in the blood stream and tissues, with subsequent undesirable toxicity and poor therapeutic efficiency (Ansel et al., 2011). These limitations have led to the concept of controlled drug delivery system; even though controlled drug delivery systems sometimes fail to achieve adequate release of the initial bolus and site specific drug delivery or may result in dose dumping. On the basis of these considerations, the bi-layer tablet was proposed (Niwa et al., 2013). This is a tablet suitable for sequential release of two drugs in combination: for the separation of two or more active pharmaceutical ingredients (APIs) from each other and to control the release of API from one layer by utilizing the functional property (such as osmotic property) of the other layer. Bi-layer tablets are also suitable for formulating sustained release tablet in which one layer is formulated to obtain immediate release of a drug with the aim of reaching a high serum concentration in a short period of time, while the second layer is designed for sustained release in order to maintain an effective plasma level of the drug for a prolonged period of time. The pharmacokinetic advantage relies on the fact that drug release from fast releasing layer leads to a sudden rise in the blood concentration while the blood level is maintained at steady state as the drug is released from the sustaining layer (Sharif et al., 2011).

Various excipients have been utilized in the formulation of bilayer tablets. For example, sodium starch glycolate (SSG) and Hydroxypropylmethyl cellulose (HPMC) have been used as superdisintegrants and sustained release polymer respectively in the formulation of bilayer tablets of glimepride and metformin hydrochloride (Reddy et al., 2010). In another study, bilayer tablets of ranitidine and aceclofenac were prepared using three different superdisintegrants (Crospovidone, Croscarmellose sodium, Sodium starch glycolate) at three different concentrations for the immediate—release layer of ranitidine while a sustained release layer containing aceclofenac was prepared using three different release-retarding polymers (Chitosan, Xanthangum, HPMC E15) (Logidhasan et al., 2013). The immediate release layer formula was observed to release 98.45% of ranitidine HCL in 10 min while the floating sustained release formulation released 98.44% of aceclofenac in 12 h.

Inspite of the advantages bilayer tablets offer, a major challenge is that it is more difficult to predict the long term mechanical properties of these delivery systems due to poor mechanical and compression characteristics of the constituent materials in the compacted adjacent layers, insufficient hardness and their tendency to delaminate at the interface between the adjacent compacted layers during and after various stages of the formulation process (Desphande et al., 2011). The use of suitable excipients such as modified starches that can impart mechanical strength to the formulation of bilayer tablets could overcome these challenges. Yams are primary agricultural commodities in many parts of Africa and South East Asia, classified as the third most important tropical root crop after cassava and potatoes (Akoroda and Hahn, 1995). Starch obtained from *Dioscorea dumetorum* (bitter yam) has been utilized as a binder at concentration of 2.5–10% w/w in chloroquine phosphate tablets to produce tablets of high mechanical strength and prolonged release in comparison to corn starch B.P., a commercial pharmacopeia starch of well-established physico-chemical properties (Okunlola and Odeku, 2011). When modified by acid hydrolysis, bitter yam starches showed potential as a directly compressible excipient for controlled release (Odeku and Picker-Freyer, 2009). In another study, white yam starch was shown to have superior disintegrant properties to corn starch and bitter yam starch (Okunlola and Odeku, 2008) when used at concentrations 5–20%w/w in chloroquine phosphate tablets. White and bitter yam starches therefore show potential as superdisintegrants and sustained–release polymers respectively in the tablet formulations. The model drug for this study is aceclofenac, molecular formula of C_16_H_13_Cl_2_NO_4_ and mass 354.2 g/mole. It is one of the non-steroidal anti-inflammatory drugs (NSAIDs) used in the symptomatic treatment of rheumatoid arthritis, osteoarthritis and ankylosing spondylitis. Aceclofenac is well absorbed from the gastrointestinal tract; peak plasma concentrations are reached 1–3 h after an oral dose (Achutha et al., 2008). The short half-life and dosing frequency make aceclofenac an ideal candidate for sustained release. Furthermore, reduction of dosing frequency of aceclofenac will minimize the risk of serious upper gastrointestinal effects such as bleeding, perforation and obstruction associated with NSAIDs.

Aceclofenac sodium has previously been formulated as bilayer tablets using mainly synthetic polymers (Karthikeyini et al., 2009; Dey et al., 2012; Sharma et al., 2014). In this study, aceclofenac was formulated as a bilayer tablet consisting of a fast release layer and the sustained layer using carboxymethylated white yam starch (CWY) and acid-hydrolyzed bitter yam starch (ABY) respectively. The fast release of aceclofenac is to ensure an immediate relief from arthritic pain, while the sustained layer ensures the maintenance of an effective level in the body.

## Materials and methods

### Materials

Aceclofenac was obtained from Indo Gulf Co., Mumbai, India. Mono-chloroacetic acid was obtained from Alfa Aesear, Massachusetts, USA. Sodium Starch Glycolate (SSG) was obtained from Patel Chem Ltd, Ahmedabad, India, and Hydroxypropylmethyl cellulose (HPMC) was obtained from Oxford Lab Chemicals, Maharashtra, India. All other reagents used were of analytical grade. Tubers of *Dioscorea rotundata* (white yam) and *Dioscorea dumetorum* (bitter yam) were obtained from local farmers in Ibadan, Nigeria and authenticated at the herbarium department of Forestry Research Institute of Nigeria, FRIN, (FHI 109672 and 109674 respectively).

### Methods

#### Extraction of yam starch

The starches were extracted from the relevant yam tubers using established methods (Young, 1984).

#### Modification of white yam starch

Forty grams of native yam starch was mixed with 2 g of sodium hydroxide and 4 g of monochloroacetic acid in a beaker. One hundred milliliters of isopropyl alcohol and 100 ml of water were added with continuous stirring to obtain homogeneity. Subsequent reaction was allowed to proceed at 5°C for 2 h. Aqueous acetic acid (50% ^v^/_v_) was added to the resulting mixture until pH 5 was obtained. The modified starch was washed with 80% ^v^/_v_ aqueous ethanol To obtain a neutral pH 7 and then dried at 50° C for 6 h. The dried starch was powdered, sieved through a 120 mesh sieve (125-μm) and then stored in an air-tight container (Singh et al., 2011).

#### Degree of substitution (DS)

Modified starch (0.5 g) was dissolved in 20 ml of 0.2 M NaOH. Distilled water (50 ml) was added and the resulting solution was transferred into a 100 ml volumetric flask and made up to volume with water. Twenty five milliliters of the solution was transferred to a flask and made up to 50 ml with distilled water. The excess of NaOH was back-titrated with 0.05 M Hcl using phenolphthalein as an indicator. The titration was done in triplicates and the mean value determined for the volume of HCl used. A blank titration was then carried out (Stojanovic et al., 2005).

(1) $$\text{D}.\text{S}.=\frac{162\times \text{nCOOH}}{\left(\text{mds}-58\right)\times \text{nCOOH}}$$

where 162 = molar mass of anhydrous glucose unit (AGU) g/mol;

mds = mass of dried sample

58 = molar mass of CH_2_COOH

nCOOH = (V_b_ - V) × C_HCL_ × 4

V_b_ = volume of HCl used for titration of blank in back titration

V = volume used in titration of sample

C_HCL_ was concentration of HCl

4 = ratio of total volume of solution taken for titration.

#### Modification of bitter yam starch

Three hundred grams (dry basis) of native bitter yam starch were hydrolysed by incubating the starch in 600 ml of a 6% Hcl solution at a temperature of 23 ± 1°C for 192 h without stirring (Atichokudomchai and Varavinit, 2003). The suspension was neutralized with 10% w/v NaOH solution, and the starch slurry was washed five times with distilled water and dried in a hot air oven at 40°C for 24 h. The starch was pulverized and then screened through a125-μm mesh sieve.

#### Characterization of starch

***Particle size and morphology***. The shape and mean particle size of 300 starch granules were determined using an optical microscope.

***Swelling power***. The swelling power of native and modified starches was determined using the method described by Bowen and Vadino (1984). Starch suspension (5% ^w^/_v_) was prepared at room temperature and shaken for 5 min. The dispersion was allowed to stand for 24 h before the sedimentation volume was measured and the swelling capacity was calculated. Determinations were done in triplicates.

***FT-IR analysis***. The native and modified starches were analyzed by FT-IR (FT-IR-Thermo Nicolet Nexus 870 Madison, WI, USA) in transmission mode. Transmission spectra were recorded using at least 64 scans with 8 cm-1 resolution in the spectral range 4000–400 cm-1.

***Determination of particle density***. The particle densities of the starches were determined by using Multivolume pycnometer 1305 (Micromeritics Inst. Corp. Norcross Georgia, USA). The bulk density of each starch powder at zero pressure (loose density) was determined by pouring 10 g of the powder at an angle of 45°C through a funnel into a glass measuring cylinder with a diameter of 21 mm and a volume of 50 ml. The tapped density was measured by applying 100 taps to 10 g of each starch sample in a graduated cylinder at a standardized rate of 38 taps per minute. Determinations were done in triplicates.

***Flowability***. The flowability of the starches was assessed using the Hausner's ratio and the Carr's index. The Hausner's ratio was determined as the ratio of the initial bulk volume to the tapped volume while Carr's index (% compressibility) was calculated as follows:

(2) $$\text{Carr}\prime \text{s Index}=\frac{\text{tapped index}-\text{bulk density}}{\text{tapped index}}\times 100$$

***Angle of repose***. An open ended cylinder of diameter 2.8 cm was placed on a base of similar diameter. 30 g of starch powder was allowed to flow through a funnel, under the force of gravity, to form a conical heap. The angle of repose was calculated from:

(3) $$\text{Tan}\theta =\frac{\text{h}}{\text{r}}$$

Where *h* is the height of powder and *r* is the radius of the base of the cone. The angle of repose was calculated from a mean of three determinations.

#### Preparation of bi-layer tablet

***Formulation of fast release layer***. The fast release layer was formulated by uniformly mixing aceclofenac sodium (50%^w^/_w_) with carboxymethylated white yam starch or sodium starch glycolate (2.5, 5.0, and 7.5% ^w^/_w_), aspartame (1%^w^/_w_), colorant (1% ^w^/_w_) and microcrystalline cellulose (MCC) to 100% ^w^/_w_. Magnesium stearate (1% ^w^/_w_) and talc (2% ^w^/_w_) were added just before blending.

***Formulation of sustained release layer***. Aceclofenac sodium sustained release layer was prepared by wet granulation method. Aceclofenac sodium (50% ^w^/_w_), acid-modified bitter yam starch or HPMC (30% ^w^/_w_), Talc (0.5% w/w), magnesium stearate (1% w/w) and Sodium carboxymethyl cellulose (in sufficient quantity to make up to 100%), were passed through a sieve (250 μm), dry mixed for 5 min in a planetary mixer (Model A120, Hobart Manufacturing Co, UK) and then moistened with PVP (10% ^w^/_w_) dissolved in appropriate amount of isopropyl glycol to produce granules. Massing was continued for 5 min and the wet masses were granulated by passing them manually through a mesh 12 sieve (1400 μm). The granules were dried in a hot air oven at 50°C for 18 h. Dried granules were screened through a mesh 16 sieve (1000 μm) and then stored in an air-tight container.

The total dose of aceclofenac for a once-daily sustained release formulation was calculated by the following (Karna, 2012):

Loading dose (D_L_) = 100 mg

To calculate the maintenance dose

D_t_ = D_L_(1 + 0.693 × t/t_1/2_)

where Dt = Total dose of drug

Dose = dose of immediate release (100 mg)

t = time (hours) during which the sustained release is desired (8 h)

t_1/2_ = half-life of aceclofenac (4 h)

D_t_ = 100[1 + 0.693 × (8/4)] = 338.6 mg = 340 mg

Therefore sustained release dose = 340 − 100 = 240 mg

***Compression of bi-layer tablet***. Granules for sustained release layer (480 mg) was compressed lightly with a predetermined load on a Carver hydraulic press (model C, Carver Inc. Menomonee Falls, WI) using a 10.5 mm die and flat-faced punch at an initial compression pressure of 56.5 Mpa for 15 s. Over this compressed layer, required quantities of formulation of the fast release layer (200 mg) was placed and final compression at 113 MNm^−2^ pressure for 15 s was done to form a bi-layer tablet.

***Evaluation of bi-layer tablets***. Twenty tablets were selected at random and their average weight was determined within ±1 mg (Mettler PC 440 Delta range®, CH-8606 Greifensee-Zurich, Switzerland). Using a micrometer screw gage, the thickness of 20 tablets was measured within ±0.01 mm.

The crushing strength of the tablets were determined at room temperature by diametral compression using a tablet hardness tester (DBK Instruments Mumbai, 400,060 model EH 01). The results were taken only from tablets which split cleanly into two halves without any sign of lamination. The percent friability of the tablets was determined using a friabilator (DBK Instruments, England) operated at 25 rpm for 4 min. All measurements were made in triplicates and the results given are the mean values.

The disintegration time of the tablets was determined in distilled water at 37 ± 0.5°C using a disintegration tester (DBK Instrument, England). Determinations were done in triplicates.

Ten tablets were crushed and dissolved in Phosphate buffer pH 6.8 and assayed for drug content using a UV/Visible Spectrophotometer (Jenway UV-7804c print, England) at wavelength 275 nm to determine the amount of aceclofenac in the tablets.

Dissolution test was carried out on the tablets using the USPXX III paddle method at 100 rpm in 900 ml of phosphate buffer pH 6.8 maintained at a temperature of at 37 ± 0.5°C for 8 h. Samples (5 ml) were withdrawn and replaced with equal amounts of fresh medium. The sample was diluted and the amount of aceclofenac released was determined at wavelength of 275 nm using a UV/Visible Spectrophotometer (Beckman Coulter DU 730 Life Science UV/Vis Spectrophotometer USA).

The results of the drug release for the formulations was fitted to First order (ln *Qt* = ln *Q*_0_ + *K*_1_*t*), Higuchi (*Q* = K_H_√t), Hixon-Crowell (*Q*^1/3^_0_ – *Q*^1/3^_*t*_ = *K t*) and Korsemeyer - Peppas (*Q*_*t*_/*Q*_∞_ = *K t^n^*) kinetic equations (Hixson and Crowell, 1931; Higuchi, 1961; Korsemeyer et al., 1983). The model of best fit was identified by comparing the values of correlation coefficients and chi-square (χ^2^) test.

## Results and discussion

### Characterization of native and modified dioscorea starches

The yields of the starches of bitter and white yam were 35% and 39% respectively. Carboxymethylated white yam of degree of substitution of 0.45 was obtained. This represents the amount of carboxymethyl groups in the molecular units of anhydrous glucose. The values of mean particle size for the native and modified starches are presented in Table 1. The native white yam starch granules were ovoid in shape with an average particle size of 12.91 ± 5.50. Modification by carboxymethylation resulted in larger particles of mean size 23.04 ± 18.61μm. Bitter yam particles were polygonal in shape with an average size of 8.53± 4.34 μm and formed aggregates on acidification to attain a greater mean particle size of 29.74 ± 14.68 μm. Particle shape can influence compaction characteristics as it is known to affect the packing behavior of starches (Marshall, 2009).

**Table 1:** **Physical and material properties of native and modified yam starches**.

**Starch**	**Particle size**	**Particle shape**	**Bulk density (g/cm^3^)**	**Tap density (g/cm^3^)**	**Carr Index (%)**	**Hausner ratio**	**Angle of repose (°)**	**Swelling power**
Native white	12.91 ± 5.50	Ovoid	0.58 ± 0.02	0.73 ± 0.03	20.55	0.7945	57.10 ± 3.10	1.67 ± 0.06
Carboxymethyl white	23.04 ± 18.61	Ovoid aggregates	0.67 ± 0.00	0.80 ± 0.04	16.25	0.8375	40.50 ± 2.90	3.35 ± 0.21
Native bitter	8.53 ± 4.34	Polygonal	0.46 ± 0.02	0.54 ± 0.02	14.81	0.8519	51.88 ± 2.25	1.50 ± 0.10
Acidified bitter	29.74 ± 14.68	Polygonal aggregates	0.69 ± 0.03	0.83 ± 0.00	29.21	0.8313	57.1 ± 3.54	1.15 ± 0.21

**Figure 1:**
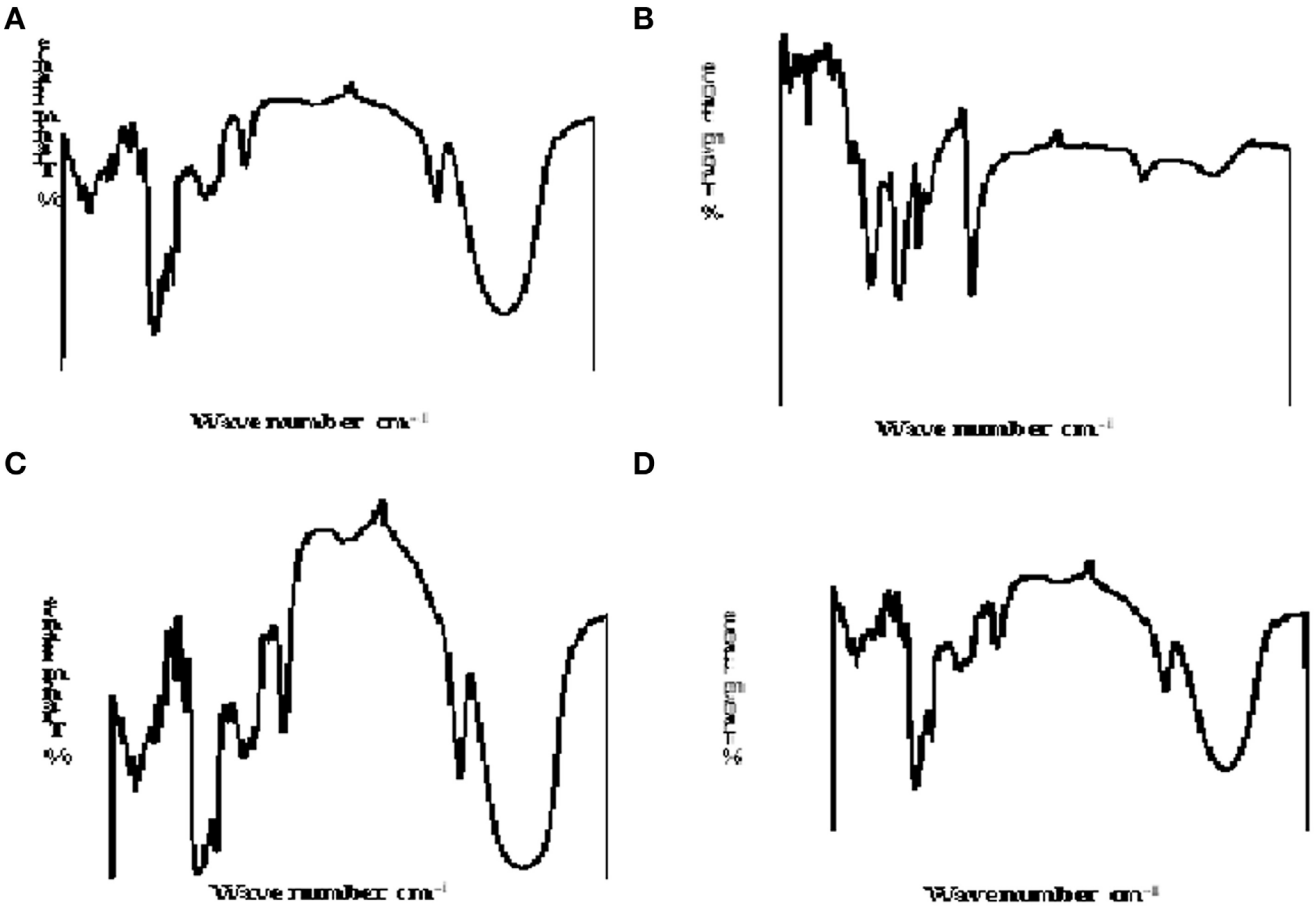
**IR spectra of (A) native white yam**. **(B)** carboxymethylated white yam starch; **(C)** native bitter yam and **(D)** acid-modified bitter yam.

The FT-IR spectra of the native and modified starches are shown in Figure 1. The FT-IR spectra of carboxymethylated starch (CWY) showed a slight modification compared to the native form as there was a reduced intensity of the absorption band at 3600 cm^−1^, due to OH stretching. This indicated that some of the OH groups were methylated. Furthermore, there was a sharp absorption band at about 1760 cm^−1^, which may be attributed to the carbonyl groups introduced into the molecule. The spectra of the modified bitter yam (ABY) showed a stretching OH (3396 cm^−1^) and a stretching CH (2929 cm^−1^) absorption bands which could be linked to acidification of alcohol groups in the starch polysaccharides.

The values of particle density, bulk density and tap density for the native and modified yam starches are presented in Table 1. From the values of the bulk and tap densities, the Carr's index and Hausner's ratio were calculated. The ranking of both bulk and tapped density was ABY> CWY> native white> native bitter. The bulk density of a starch powder describes its packing behavior while the tap density indicates the rate and extent of packing that would be experienced by the material during the various unit operations of tableting. Higher bulk density is advantageous in tableting because of a reduction in the fill volume of the die. The Carr's index is a measure of the flowability and compressibility of a powder and the ranking was native white> ABY > CWY > native bitter. The lower the Carr index the better the flowability but the poorer the compressibility. This result indicates that modification of bitter yam by acid-hydrolysis produced starches of better compressibility. On the other hand, modification of white yam starch by carboxymethylation resulted in improved flowability. The Hausner's ratio (tap to bulk density) provides an indication of the degree of densification and the values for the obtained for the starches were in the same rank order.

Modification of native white by carboxymethylation resulted in a marked increase (*p* < 0.05) in swelling power. This is due to the higher hydrophilicity of the carboxyl group which takes up more of water resulting in greater swelling. This is in contrast to bitter yam in which acidification resulted in a decreased swelling power due to the introduction of the more hydrophobic carbonyl group which may have reduced its affinity for water.

### Evaluation of bi-layer tablets of aceclofenac

The different formulations that were used to prepare the immediate- and sustained- release layers of the bi-layer tablets of aceclofenac are presented in Table 2. Batches 1–12 were obtained from combinations of the immediate and sustained layer formulations and are presented in Table 3. The mechanical and release properties of the bi-layer tablets are also presented in Table 3. All the formulated tablets passed weight variation test as the percentage weight variation was within the United State Pharmacopeia limits of ±5% of the weight. The average thickness of all the formulations was found to be within the limit of specifications. Generally, it was observed that formulations containing ABY produced tablets with significantly (*p* < 0.05) higher crushing strength than those containing HPMC. Crushing strength increased with increase in CWY in all formulations. On the other hand, tablets containing ABY generally gave significantly (*p* < 0.05) lower friability values with friability values decreasing with increase in CWY concentration. It was observed that friability was more than 1% in formulations containing SSG while those containing CWY had lower values. Mechanical strength is of importance in tablets as this determines the extent to which the tablets can withstand the rigors of packaging, transportation and handling.

Table 2**Formulations for the immediate- and sustained-release layers of the bi-layer tablets of aceclofenac**.

**Table:** 

**Fast Release Layer**						
**Ingredient**	**F1 (mg)**	**F2 (mg)**	**F3 (mg)**	**F4 (mg)**	**F5 (mg)**	**F6 (mg)**
Aceclofenac	100	100	100	100	100	100
Carboxymethylated white yam starch	5	10	15	–	–	–
Sodium starch glycollate	–	–	–	5	10	15
Aspartame	2	2	2	2	2	2
Talc	1	1	1	1	1	1
Magnesium stearate	2	2	2	2	2	2
Microcrystalline cellulose (MCC) to	200	200	200	200	200	200

**Table:** 

**Sustained Release Layer**						
**Ingredient**			**S1 (mg)**			**S2 (mg)**
Aceclofenac			240			240
Acetylated bitter yam starch			144			–
HPMC			–			144
PVP			48			48
Sodium carboxymethyl cellulose			40.8			40.8
Talc			2.4			2.4
Magnesium stearate			4.8			4.8

**Table 3:** **Mechanical and release properties of bi-layer tablets of aceclofenac (mean ± standard deviation, *n* = 3)**.

**Batch**	**Formulation**	**Average weight (g)**	**Tablet thickness (mm)**	**Crushing strength (MNm^−2^)**	**Friability (%)**	**Disintegration time (min)**	**t_90_ (min)**
1	F1S1	0.68 ± 0.01	5.09 ± 0.09	147.90 ± 11.05	0.75 ± 0.02	24.00 ± 1.55	450 ± 11.55
2	F2S1	0.67 ± 0.01	5.05 ± 0.05	152.70 ± 17.20	0.62 ± 0.05	25.00 ± 2.20	445 ± 12.20
3	F3S1	0.68 ± 0.00	5.06 ± 0.07	165.73 ± 11.25	0.44 ± 0.10	29.00 ± 1.00	410 ± 10.70
4	F4S1	0.66 ± 0.00	5.05 ± 0.05	120.13 ± 15.27	1.22 ± 0.03	27.00 ± 0.95	360 ± 13.95
5	F5S1	0.68 ± 0.01	5.07 ± 0.10	136.03 ± 11.75	1.16 ± 0.08	23.00 ± 2.50	310 ± 8.50
6	F6S1	0.68 ± 0.01	5.03 ± 0.09	150.13 ± 8.80	1.12 ± 0.04	28.00 ± 3.56	300 ± 7.56
7	F1S2	0.67 ± 0.02	5.10 ± 0.08	73.87 ± 12.14	1.45 ± 0.06	30.00 ± 3.30	420 ± 13.30
8	F2S2	0.68 ± 0.02	5.09 ± 0.21	88.50 ± 11.54	1.36 ± 0.07	33.00 ± 2.44	350 ± 12.44
9	F3S2	0.67 ± 0.00	5.10 ± 0.10	96.03 ± 7.75	1.20 ± 0.08	40.00 ± 3.55	330 ± 9.55
10	F4S2	0.69 ± 0.01	5.08 ± 0.09	68.13 ± 4.50	1.62 ± 0.04	36.00 ± 2.45	460 ± 8.45
11	F5S2	0.68 ± 0.00	5.09 ± 0.09	73.87 ± 12.14	1.75 ± 0.06	32.00 ± 2.75	420 ± 8.75
12	F6S2	0.67 ± 0.002	5.11 ± 0.21	88.50 ± 11.54	1.86 ± 0.01	40.00 ± 4.50	360 ± 14.50

The immediate release layer in all tablets disintegrated rapidly within a period of ≤3 min. it was observed that tablets containing HPMC in the sustained release layer absorbed water and were swollen for a considerable period of time before disintegrating. The gelling of HPMC could have retarded water penetration into the tablets resulting in increase in disintegration times (DT) as shown in Table 3. Generally, DT increased with concentration and crushing strength for the tablets containing CWY as superdisintegrant. However, for tablets containing SSG, DT appeared to reduce with increase in concentration from 2.5 to 5%w/w and then increased significantly (*p* < 0.05) when concentration was increased to 7.5%w/w. Generally tablets containing the *Dioscorea* starches gave faster disintegration times. Disintegration of tablets play a vital role in the dissolution process since it determines to a large extent the area of contact between the solid and liquid (Odeku and Itiola, 2003).

**Figure 2:**
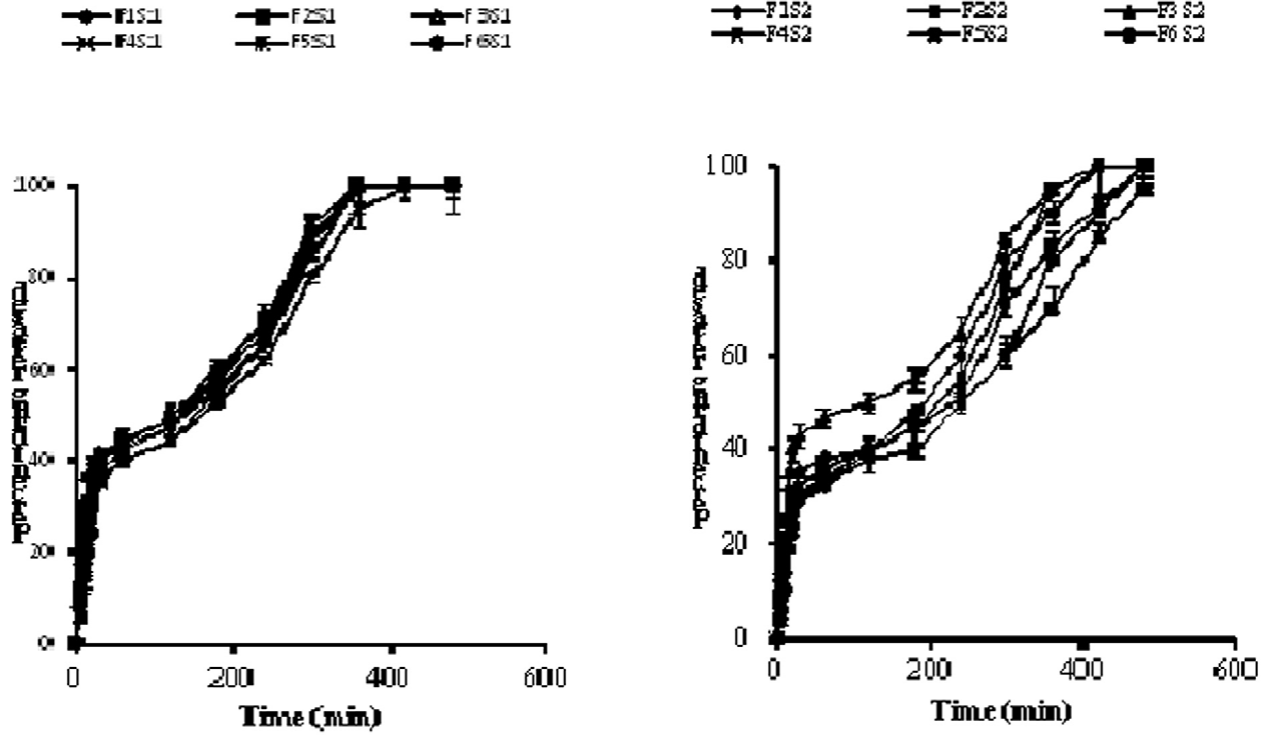
**Plot of cumulative percent of aceclofenac sodium released vs. time (min)**.

Assay of drug content revealed 98.9% of aceclofenac. The plots of cumulative drug release vs. time are shown in Figure 2. The dissolution plots showed there was an initial rapid release of the drug (≤40% of the total amount of aceclofenac) attributed to the dissolution of the immediate-release portion of the bilayer tablets. After a period of about 20 min, the sustained-release portion of the tablets was observed to release aceclofenac sodium in a controlled manner. From the plots obtained, the time taken for 90% of drug release (t_90_) was determined. Dissolution times (t_90_) were within a range of 300 ± 7.56–4.0 ± 12.45 min. Dissolution time decreased with increase in concentrations of the two superdisintegrants from 2.5 to 7.5%w/w for all formulations. Generally, tablets containing HPMC with SSG as superdisintegrant gave longer dissolution times than similar tablets containing CWY. HPMC in the presence of water forms a gel which can further increase the diffusion path length of the drug. The viscous nature of the gel can affect the diffusion coefficient of the drug thereby prolonging time for drug release (Gupta et al., 2002). Formulations containing both CWY and ABY (formulations 1, 2, and 3) appeared to provide adequate balance between mechanical strength and adequate sustained drug release.

The kinetics of drug release is important due to their influence on drug bioavailability. The physicochemical properties of drug and polymer have been shown to govern the release of drug from formulations which could also modify their release kinetics (Nayak et al., 2010). Data obtained from *in vitro* release studies were fitted to various kinetic equations to determine the kinetics and mechanism (s) of drug release from the bilayer tablet formulations. The correlation coefficient (*R*^2^) and chi-square (χ^2^) test were employed as error analysis methods to determine the best-fitting equation (Chowdhury and Das Saha, 2011) and the results are presented in Table 4. The chi-square test appeared to be a better method to determine the best-fitting model because experimental values are compared with those calculated from the model on the same abscissa and ordinate. *In vitro* dissolution kinetics for the bilayer tablets generally followed the Higuchi and Hixson-Crowell models. Higuchi describes drug release as a diffusion process that is square root time dependent. Hixson-Crowell applies to pharmaceutical dosage forms, such as tablets, where the dissolution occurs in planes that are parallel to the drug surface if the tablet dimension diminishes proportionally, in such a manner that the initial geometric form keeps constant all the time (Costa and Sousa Lobo, 2001). Diffusion, swelling and erosion are the three most important rate-controlling mechanisms in sustained release formulations. Even though drug release from the polymeric systems is mostly best described by fickian diffusion, for certain formulations containing swelling polymers such as HPMC, other processes include relaxation of polymer chain and inhibition of water which causes the polymer to swell thereby reverting from an initial glassy to a rubbery state (Ritger and Peppas, 1987).

**Table 4:** **Kinetics of *in vivo* release from bilayer tablets of aceclofenac sodium**.

**Batch**	**First order**		**Higuchi**		**Hixson-Crowell**		**Korsemeyer-Peppas**	
	**R^2^**	**x^2^**	**R^2^**	**x^2^**	**R^2^**	**x^2^**	**R^2^**	**x^2^**
F1S1	0.8201	1.022	0.9555	0.061	0.8557	0.000	0.7933	1.413
F2S1	0.8210	0.380	0.9685	0.195	0.9374	0.290	0.9163	0.136
F3S1	0.8728	0.043	0.9485	0.011	0.8856	0.400	0.8926	0.273
F4S1	0.8605	0.060	0.9714	0.027	0.8856	0.089	0.8422	0.455
F5S1	0.8364	0.978	0.9563	0.021	0.9029	0.011	0.8536	1.355
F6S1	0.8687	0.755	0.9711	0.055	0.9259	0.040	0.8797	1.519
F1S2	0.8446	0.309	0.9335	0.064	0.9269	0.004	0.9154	0.491
F2S2	0.7384	0.159	0.9389	0.150	0.9068	0.068	0.9272	0.998
F3S2	0.8201	0.034	0.9399	0.069	0.8557	0.386	0.9207	1.063
F4S2	0.8211	0.448	0.9470	0.080	0.9374	0.325	0.8985	0.827
F5S2	0.8730	0.322	0.9470	0.065	0.9183	0.002	0.9340	0.660
F6S2	0.8605	0.033	0.9500	0.243	0.8856	0.134	0.9255	0.994

## Conclusion

Formulation of bilayer tablets of aceclofenac using carboxymethylated white yam starch as superdisintegrant and acidified bitter yam starch as sustained-release polymers produced tablets of better mechanical strength than those containing the standard SSG and HPMC. Furthermore, tablets containing the modified Dioscorea starches gave faster disintegration times and prolonged drug release. Carboxymethylated white yam and acidified bitter yam starches could therefore be used as alternative excipients in the formulation of bilayer tablets of drugs that require high mechanical strength (to minimize the problems of capping and lamination) for immediate and sustained release of medicaments.

### Conflict of interest statement

The author declares that the research was conducted in the absence of any commercial or financial relationships that could be construed as a potential conflict of interest.

## Abbreviations

ABY: Acid-modified bitter yam starch
CWY: Carboxymethylated white yam starch.

## References

[B1] Achutha N. U. Srinivas M. Meka S. R. Averineni K. R. Pralhad K. Nayanabhirama U. (2008). Preparation and, *in vitro*, preclinical and clinical studies of aceclofenac spherical agglomerates. Euro. J. Pharm. Biopharm. 70, 674–683. 10.1016/j.ejpb.2008.06.010 18606224

[B2] Akoroda M. O. Hahn S. K. (1995). Yams in Nigeria: status and trends. Afr. J. Root Tuber Crops 1, 38–41.

[B3] Ansel H. C. Allen L. V. Jr. Popovich N. G. (2011). Pharmaceutical Dosage Forms and Drug Delivery Systems, 9th Edn. Philadelphia, PA: Lippincott Williams & Wilkins.

[B4] Atichokudomchai N. Varavinit S. (2003). Characterization and utilization of acid-modified cross linked tapioca starch in pharmaceutical tablets. Carbohydr. Polym. v. 53, 263–270 10.1016/S0144-8617(03)00070-5

[B5] Banker G. S. Anderson N. R. (1986). Tablets, in The Theory and Practice of Industrial Pharmacy, 3rd Edn., eds Lachman L. Lieberman H. A. Kanig J. L. (Philadelphia, PA: Lea and Febiger), 301–303.

[B6] Bowen F. E. Vadino W. A. (1984). A simple method for differentiating sources. Drug Deve. Ind. Pharm. v. 10, 505–551 10.3109/03639048409041403

[B7] Chowdhury S. Das Saha P. (2011). Comparative analysis of linear and non-linear methods of estimating the pseudo-second-order kinetic parameters for sorption of Malachite Green onto pretreated rice husk. Bioremediation J. 15, 181–188 10.1080/10889868.2011.624140

[B8] Costa P. Sousa Lobo J. M. (2001). Modeling and comparison of dissolution profiles. Euro. J. Pharm. Sci. 13, 123–133. 10.1016/S0928-0987(01)00095-1 11297896

[B9] Desphande R. D. Gowda D. V. Mohammed N. Mramwar D. N. (2011). Bilayer tablets—anemerging trend: a review. Int. J. Pharm. Sci. Res. 2, 2534–2544.

[B9a] Dey S. Mahanti B. Khila S. Mazumder B. Gupta S. D. (2012). Formulation development and optimization of bilayer tablets of aceclofenac. Expert Opin. Drug. Deliv. 9, 1041–1050. 10.1517/17425247.2012.707187 22788786

[B10] Gupta P. Vermani K. Garg S. (2002). Hydrogels: from controlled release to PH responsive drug delivery. Drug Discov. Tech. 7, 569–579. 10.1016/S1359-6446(02)02255-9 12047857

[B11] Higuchi T. (1961). Rate of release of medicaments from ointment bases containing drugs in suspension. J. Pharm. Sci. 50, 874–875. 10.1002/jps.2600501018 13907269

[B12] Hixson A. W. Crowell J. H. (1931). Dependence of reaction velocity upon surface and agitation. Ind. Eng. Chem. 23, 923–931 10.1021/ie50260a018

[B17] Karna N. (2012). Design, development and evaluation of novel sustained release bi-layer tablets of lornoxicam based on the combination of hydrophilic matrix formers and basic pH modifiers. Int. J. Pharm. Biol. Sci. 3, 392–402. 19895367 10.3109/10837450903059371

[B17a] Karthikeyini S. C. Jayaprakash S. Abirami A. Halith S. M. (2009). Formulation and evaluation of aceclofenac sodium bilayer sustained release tablets. Int. J. Chemtech Res. 1, 1381–1385.

[B14] Korsemeyer R. W. Gurny R. Doelker E. M. Buri P. Peppas N. A. (1983). Mechanism of solute release from porous hydrophilic polymers. Int. J. Pharm. 15, 25–35 10.1016/0378-5173(83)90064-9

[B15] Logidhasan L. Suresh S. Mathivanan M. (2013). Formulation and *in vitro* evaluation of bilayer floating tablets of Aceclofenac and Ranitidine Hcl Int. Res. J. Pharm. App. Sci. 3, 88–94.

[B16] Marshall K. (2009). Compression and consolidation of powdered solids, in The Theory and Practice of Industrial Pharmacy, eds Lachman L. Lieberman H. A. (New Delhi: CBS Publishers and Distributors), 113–170.

[B18] Nayak A. K. Hasnain M. S. Beg S. Alam M. I. (2010). Mucoadhesive beads of gliclazide: design, development and evaluation. Sci. Asia 36, 319–325 10.2306/scienceasia1513-1874.2010.36.319

[B19] Niwa M. I. Hiraishi Y. Iwasaki N. Terada K. (2013). Quantitative analysis of the layer separation risk in bilayer tablets using terahertz pulsed imaging. Int. J. Pharm. 452, 249–256. 10.1016/j.ijpharm.2013.05.010 23680735

[B20] Odeku O. A. Itiola O. A. (2003). Evaluation of the effects of khaya gum on the mechanical and release properties of paracetamol tablets. Drug Dev. Ind. Pharm. 29, 311–320. 10.1081/DDC-120018205 12741612

[B21] Odeku O. A. Picker-Freyer K. M. (2009). Characterization of acid modified *Dioscorea* starches as direct compression excipient. Pharm. Dev. Technol. 14, 259–270. 10.1080/10837450802572367 19519180

[B22] Okunlola A. Odeku O. A. (2008). Comparative evaluation of starches from Dioscorea species as intragranular tablet disintegrant. J. Drug Deliv. Sci. Technol. 18, 444–447 10.1016/S1773-2247(08)50085-2

[B23] Okunlola A. Odeku O. A. (2011). Evaluation of starches obtained from four *Dioscorea* species as binding agent in chloroquine phosphate tablet formulations. Saudi Pharm. J. 19, 95–105. 10.1016/j.jsps.2011.01.002 23960747 PMC3744955

[B24] Reddy S. Panakanti P. K. Kandaghatla R. Yamsani M. R. (2010). Formulation and release characteristic of a bilayer matrix tablet containing glimepiride immediate release component and metformin hydrochloride as sustained release component. Int. J. Pharm. Sci. Nanotechnol. 3, 851–859.

[B25] Ritger P. L. Peppas N. A. (1987). A simple equation for description of solute release II. Fickian and anomalous release from swellable devices. J. Control Rel. 5, 37–42. 10.1016/0168-3659(87)90035-6 25356469

[B26] Sharif A. E-Rabbani M. Akhtar M. F. Akhtar B. Saleem A. Murtaza G. (2011). Design and evaluation of modified release bilayer tablets of flurbiprofen. Adv. Clin. Exp. Med. 20, 343–349.

[B26a] Sharma R. Mittal S. Kumar D. Singla P. Garg V. Arora A. (2014). Formulation and evaluation of bi-layer tablet of Aceclofenac sodium and Tramadol hydrochloride. J. Fundam. Pharm. Res. 2, 22–34.

[B27] Singh A. V. Nath L. K. Guha M. Kumar R. (2011). Microwave assisted synthesis and evaluation of cross-linked carboxymethylated Sagi starch as superdisintegrant. J. Pharm. Pharm. 2, 42–46 10.4236/pp.2011.21005

[B13] Stojanovic Ž. Jeremić K. Jovanović S. Lechner M. D. (2005). Comparison of some methods for determination of the degree of substitution of carboxymethyl starch. Starch - Stärke 57, 79–83 10.1002/star.200400342

[B28] Young A. H. (1984). Fractionation of starch, in Starch Chemistry. Technology, 2nd Edn., eds Whistler R. L. Bemiller J. N. Pashall E. F. (London: Academic Press), 249–283.

